# Developing a strategic action plan for reducing the burden of stroke in Africa: report of the first African Stroke Leaders' Summit

**DOI:** 10.3389/fstro.2025.1555554

**Published:** 2025-02-25

**Authors:** Rufus Akinyemi, Paul Olowoyo, Stephanie Jones, Olaleye Adeniji, Gabriel Ogunde, Joseph Spencer, Foad Abd-Allah, Albert Akpalu, Liz Lightbody, Joseph Yaria, Fred S. Sarfo, Pamela Naidoo, Sarah Belson, Ad Adams Ebenezer, Ahmed Nasreldein, Akintomiwa Makanjuola, Deanna Saylor, Stanley Zimba, Lucia Ojewale, Daniel Youkee, Thierry Adoukonou, Akinkunmi Okekunle, Benjamin Anyanwu, Njideka Okubadejo, Kathleen Bateman, Rita Melifonwu, Reginald Obiako, Oyedunni Arulogun, Kolawole W. Wahab, Philip Adebayo, Patty Francis, Paul Ossu-Nguiet, Augustina Charway-Felly, Godwin Ogbole, Shamsideen Ogun, Richard Walker, Mehari Gebreyohanns, Peter Langhorne, Bo Norrving, Bruce Ovbiagele, Rajesh N. Kalaria, Adesola Ogunniyi, Caroline Leigh Watkins, Mayowa Owolabi

**Affiliations:** ^1^Institute for Advanced Medical Research and Training, College of Medicine, University of Ibadan, Ibadan, Nigeria; ^2^Centre for Genomic and Precision Medicine, College of Medicine, University of Ibadan, Ibadan, Nigeria; ^3^Department of Neurology, University College Hospital, Ibadan, Nigeria; ^4^Department of Medicine, Federal Teaching Hospital, Ido-Ekiti, Nigeria; ^5^Afe Babalola University, Ado-Ekiti, Nigeria; ^6^University of Central Lancashire, Preston, United Kingdom; ^7^Department of Neurology, Cairo University Hospital, Cairo, Egypt; ^8^University of Ghana Medical School/Korle Bu Teaching Hospital, Accra, Ghana; ^9^Kwame Nkrumah University of Science and Technology, Kumasi, Ghana; ^10^Komfo Anokye Teaching Hospital, Kumasi, Ghana; ^11^Heart and Stroke Foundation South Africa, Cape Town, South Africa; ^12^University of the Western Cape, Cape Town, South Africa; ^13^World Stroke Organization, Geneva, Switzerland; ^14^Stroke Association, London, United Kingdom; ^15^Stroke Association Support Network-Ghana (SASNET-GHANA), Accra, Ghana; ^16^Department of Neurology and Psychiatry, Assiut University, Assiut, Egypt; ^17^Department of Neurology, Johns Hopkins University School of Medicine, Baltimore, MD, United States; ^18^Department of Internal Medicine, University Teaching Hospital, Lusaka, Zambia; ^19^Faculty of Nursing, College of Medicine, University of Ibadan, Ibadan, Nigeria; ^20^School of Life Course and Population Health Sciences, King's College London, London, United Kingdom; ^21^Department of Neurology, University of Parakou, Parakou, Benin; ^22^Seoul National University, Seoul, Republic of Korea; ^23^Regions Neuroscience Hospital, Owerri, Nigeria; ^24^Department of Medicine, University of Lagos/Lagos University Teaching Hospital, Lagos, Nigeria; ^25^Neurology Unit Groote Schuur Hospital, Cape Town, South Africa; ^26^Stroke Action Nigeria, Ime Obi Ogbeoza, Onitsha, Nigeria; ^27^Department of Medicine, Ahmadu Bello University Teaching Hospital Zaria, Zaria, Nigeria; ^28^Department of Health Promotion and Education, College of Medicine, University of Ibadan, Ibadan, Nigeria; ^29^Department of Medicine, College of Health Sciences, University of Ilorin, Ilorin, Nigeria; ^30^Aga-Khan University, Medical College East Africa, Dar es Salaam, Tanzania; ^31^Neurological Association of South Africa (NASA), Johannesburg, South Africa; ^32^Department of Neurology and Stroke Unit, University Hospital of Brazzaville, Brazzaville, Republic of Congo; ^33^Neurology Department, 37 Military Hospital, Accra, Ghana; ^34^Department of Radiology, College of Medicine, University of Ibadan, Ibadan, Nigeria; ^35^Lagos State University College of Medicine, Ikeja, Lagos, Nigeria; ^36^Lagos State University Teaching Hospital, Ikeja, Lagos, Nigeria; ^37^Department of Medicine, North Tyneside General Hospital, North Shields, United Kingdom; ^38^University of Texas Southwestern, Dallas, TX, United States; ^39^University of Glasgow, Glasgow, United Kingdom; ^40^Glasgow Royal Infirmary, Glasgow, United Kingdom; ^41^Lund University, Lund, Sweden; ^42^Weill Institute for Neurosciences, School of Medicine, University of California, San-Francisco, San Francisco, CA, United States; ^43^Clinical and Translational Research Institute, Newcastle University, Newcastle, United Kingdom

**Keywords:** Africa, stroke burden, Leaders' Summit, strategic action plan, African, report

## Abstract

**Introduction:**

Stroke is a leading cause of adult neurologic disability, cognitive decline, and death worldwide, and particularly in Africa. Stroke research in Africa has exposed challenges militating against the translation of research evidence into practice and policy. The evidence-based, context-sensitive multilevel strategies required to surmount these challenges are presented in this report on the first African Stroke Leaders' Summit (ASLS) organized to tackle the burden of stroke in Africa.

**Methods:**

The Africa–UK Stroke Partnership (AUKSP) Project had a Steering Committee (SC) and four theme-based Working Groups (WGs): stroke services, stroke training/capacity building, research and stroke advocacy, each with defined terms of reference. These groups generated 20 priorities (five per thematic area) during breakout sessions at the first ASLS which were further refined into four topmost priorities (one per thematic area) at the general consensus session.

**Results:**

The topmost priorities included promoting the development of acute stroke services (stroke services), strengthening population-based stroke education focusing on prevention and symptom recognition (stroke training), research on hypertension control to reduce stroke risk (stroke research), and developing national stroke action plans (advocacy).

**Conclusion:**

Sustained reduction of stroke burden in Africa requires the adaptation of best practices to the African context, building the capacity of African stroke care professionals and using available resources with political support. Improving stroke literacy in African communities is a complementary strategy to reinforce healthy lifestyle choices and improve screening and detection of hypertension and other modifiable stroke risk factors. This process will culminate in a strategic African Stroke Action Plan (ASAP), the blueprint for the control of stroke in Africa.

## 1 Background

Stroke is a leading cause of neurologic disability, death, and dementia worldwide (Feigin et al., 2019). In Africa, stroke records an annual incidence rate of up to 316 per 100,000 person-years, a prevalence rate of up to 1,460 per 100,000 person-years, and a 3-year fatality rate reaching 84% (Owolabi, 2011; Okekunle et al., 2023; Akinyemi et al., 2021a). The burden of stroke in Africa is, therefore, among the highest in the world with a younger age of onset, and stroke mortality that is at least five times higher in sub-Saharan African (SSA) compared to high-income countries. Although significant progress has been made in understanding the epidemiology, genetics, and outcomes of stroke in Africa through research data, important gaps remain. Furthermore, stroke care has huge cost implications for patients and caregivers with funding for care primarily from out-of-pocket expenditure (Iseko et al., 2018). The impact of stroke in Africans is particularly devastating as a significant proportion of patients are adults at an active and productive stage of life with grave implications for the individual, family, and society (Connor et al., 2007). Moreover, severity, length of hospital stay, and multi-morbidities increase the direct and indirect costs of stroke care (Kaur et al., 2014). Tackling the escalating burden of stroke in Africa, therefore, requires context-sensitive multilevel strategies (Owolabi, 2011). The plethora of challenges affecting the effective prevention, management and control of stroke in Africa was the reason for the brainstorming sessions by stakeholders in the value chain of stroke care in Africa with a goal to identify barriers, opportunities, and, importantly, Africa-led strategies and solutions to reduce the burden of stroke in Africa.

The African Stroke Organization (ASO) was inaugurated in October 2020 with the primary goal of reducing the burden of stroke in Africa (Akinyemi and Brainin, 2021; Akinyemi et al., 2021b). To operationalize the vision of the ASO, the Africa–UK Stroke Partnership (AUKSP) Project was established with a networking grant provided by the UK Academy of Medical Sciences within the remit of the UK Global Challenges Research Fund (GCRF). The overarching goal of the project was to map the current landscape and facilitate the development of a strategic action plan for reducing the burden of stroke in Africa. The specific objectives were (a) to conduct an online survey to evaluate the current state of stroke care, research, training, and advocacy in Africa and (b) to conven a strategic African Stroke Leaders' Summit (ASLS) to evaluate data accrued from the online survey, (c) identify gaps, and determine priorities for formulating an African Stroke Strategic Action Plan.

## 2 Methods

### 2.1 Overall approach

The governance framework of the AUKSP Project consisted of a Steering Committee (SC) and Working Groups (WGs) based on the four thematic areas of the conceptual framework of the ASO (Akinyemi et al., 2021b). The WGs included stroke services, stroke training/capacity, research, and stroke advocacy, each with defined terms of reference (Table 1). A central WG oversaw the harmonization of the findings and recommendations of the other four WGs and leads the process of developing an African Stroke Strategic Action Plan.

**Table 1:** AUKSP working groups terms of reference.

**Stroke services WG objectives**
a) Establish current level of stroke services across Africa using the framework of the WSO Global Stroke Services Guidelines and Action Plan (Minimal, Essential, Advanced) and Lancet Commission for Stroke in LMICs. Includes Surveillance, Prevention (primary and secondary), Acute Stroke Services (including pre-hospital and hospital care) and Rehabilitation and Long-term Care (institution-based, home-based and support services) b) Set targets and priorities for 2030 for expanding stroke services and ensuring that all stages of adequate stroke care c) Set strategies and framework for adapting and operationalizing WSO guidelines for stroke care in Africa d) Set benchmarks and algorithms for monitoring and evaluating progress across African countries
**Stroke training/capacity building WG objectives**
a) Establish current state of stroke training/capacity building. Training programmes for multidisciplinary stroke care providers (Physicians, Surgeons, Nurses, Physiotherapists, Pharmacists, Laboratory Scientists, Speech and Language Therapists, Occupational Therapists, Clinical Psychologists, Dietitians, Social Workers, Health Educators etc). Research training may cover basic, clinical, translational, behavioral, and population research, with due attention to cultural sensitivities. Virtual training may include participation in the World Stroke Academy b) Set targets and priorities for 2030 for expanding stroke training/capacity building across the continuum of stroke care, including context-sensitive implementation science and translational stroke research (T1–T5) c) Set benchmarks and algorithms for monitoring and evaluating progress
**Stroke research WG**
a) Establish the current state of stroke research in Africa covering stroke epidemiologic surveillance; regional/national stroke registries; genomic, multi-omics, clinical trials across the trajectory of stroke care, stroke prevention, post-stroke vascular cognitive impairment, post-stroke outcomes and complications, quality of life, stroke services research, stroke recovery and rehabilitation, community stroke care b) Set targets and priorities for 2030 for expanding stroke research across the continuum of stroke care, including context-sensitive implementation science and translational stroke research (T1–T5) c) Set benchmarks and algorithms for monitoring and evaluating progress
**Stroke advocacy/ community engagement**
a) Establish current state of stroke awareness and advocacy. To evaluate stroke education across the lifespan and the socio-ecological framework. Evaluate African stroke support organizations (SSOs) and life after stroke support programmes. Assess programmes of engagement involving school children, inclusion of stroke education in school curricula, engagement with faith-based organizations and community leaders and gatekeepers. Assess advocacy with policy makers and governments and their stroke research, prevention, care and rehabilitation programmes b) Set targets and priorities for 2030 for expanding stroke advocacy and across the continuum of stroke care c) Set benchmarks and algorithms for monitoring and evaluating progress
**Central WG**
For harmonization/priority setting a) Harmonize the findings and recommendations of all the four-working group b) Lead the way in developing the strategic action plan for stroke

Before the stroke leaders' summit (ASLS), the WGs evaluated the current state of stroke in Africa. The WGs reviewed the extant literature on stroke in Africa (Cole et al., 2021; Sarfo et al., 2021; Owolabi et al., 2015), reviewed African datasets in the published work of the World Stroke Organization (WSO)–Lancet Neurology Commission on Stroke (Owolabi et al., 2021), and from the global survey of stroke support organizations (SSOs) (Thombs et al., 2022). Working through the datasets accrued from both the situational analysis and the online surveys (stroke expert and stroke unit), the WGs met by videoconferencing every month, and generated results using a tabulated framework based on their respective terms of reference. The tables included information on (a) the domain of stroke operation (b) Global Standards WSO guidelines (Mead et al., 2023) 2014; European Stroke Action Plan (ESAP) 2018–2030 (Norrving et al., 2018), (c) Situational Analysis in Africa, (d) 2030 Targets for Africa, (e) Key Action Steps, (f) Key Performance Indicators, and (g) Monitoring and Evaluation Framework (Gopichandran and Krishna, 2013). This body of information highlighted implementation gaps in the four thematic areas regarding stroke in Africa. These thematic areas provided the templates for WG breakout sessions during the (ASLS).

**Figure 1:**
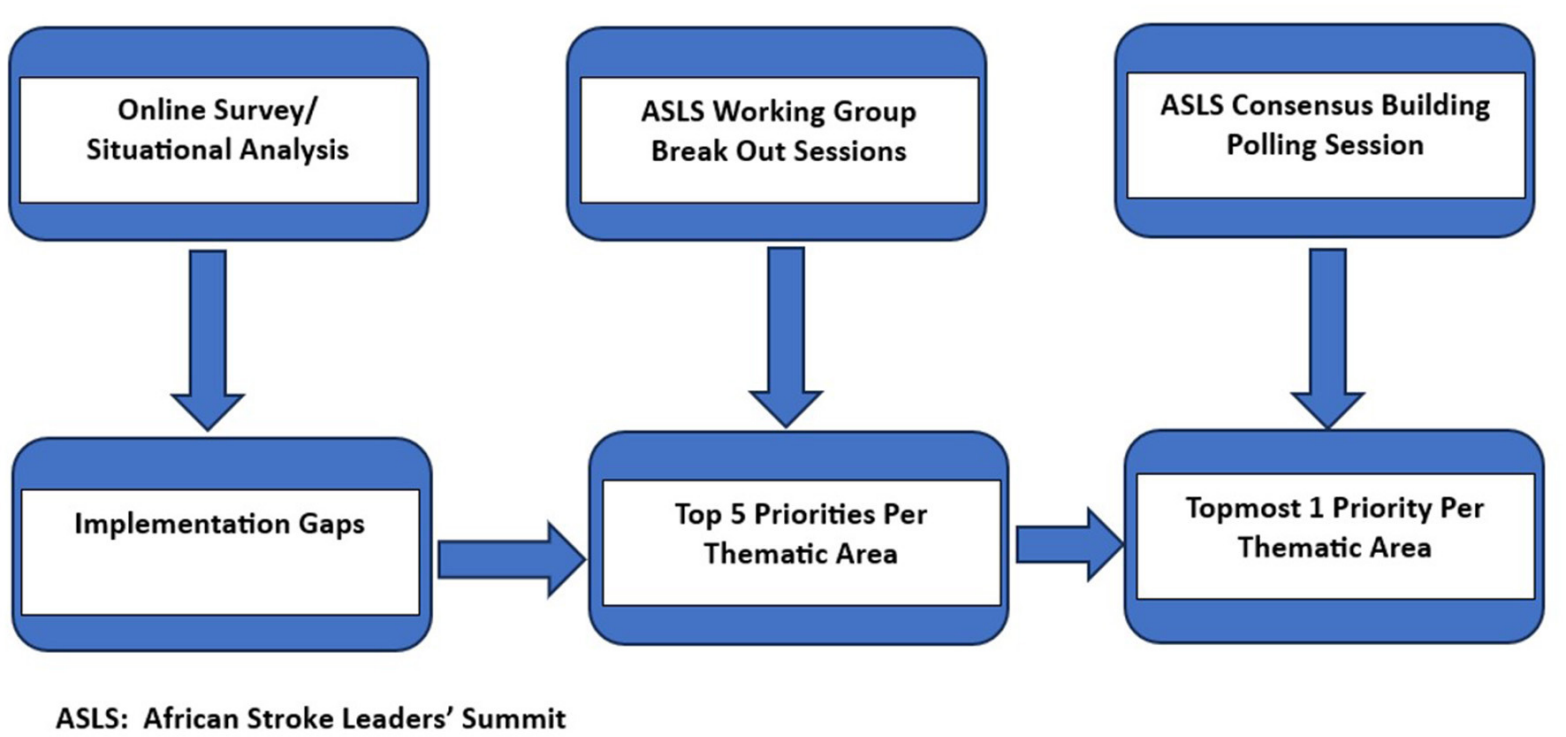
African Stroke Leaders' Summit (ASLS) workflow chart.

A 2-day ASLS took place in Ibadan, Nigeria on the 22nd and 23rd of June, 2022. The hybrid platform enabled the participation of stroke leaders from Africa and the United Kingdom. The objectives of the ASLS included (1) reviewing available evidence and survey results related to the four WGs (2) discussing and reaching a consensus on five key priorities, and (3) then ranking these to inform an action plan in each of the four thematic areas in line with the ASO conceptual framework (Akinyemi et al., 2021b). Figure 1 shows the general workflow chart of the (ASLS).

### 2.2 Participants

#### 2.2.1 Working group participants

Each WG had a chair, a co-chair, and a secretary. The membership size and professional backgrounds of the working groups are as follows:

a. *Stroke services* (12 members including stroke neurologists, stroke nurse specialists, neuro interventionists, and neuro physiotherapists).
b. *Stroke training/capacity building* (14 members including stroke neurologists, stroke nurse specialists, neurosurgeon, and clinical epidemiologist)
c. *Stroke research* (12 members including stroke neurologists, a geriatrician, stroke nurse specialists, and a neuropathologist)
d. *Stroke advocacy* (12 members including neurologists, stroke nurse specialists, stroke survivors, clinical psychologist/public health specialist, stroke rehabilitation expert, speech and language expert, neuroradiologist, and stroke public health strategist).

The WG members met monthly by videoconferencing and their meetings culminated in WG reports which were presented by the working group leaders during the ASLS.

#### 2.2.2 Summit participants

**Figure 2:**
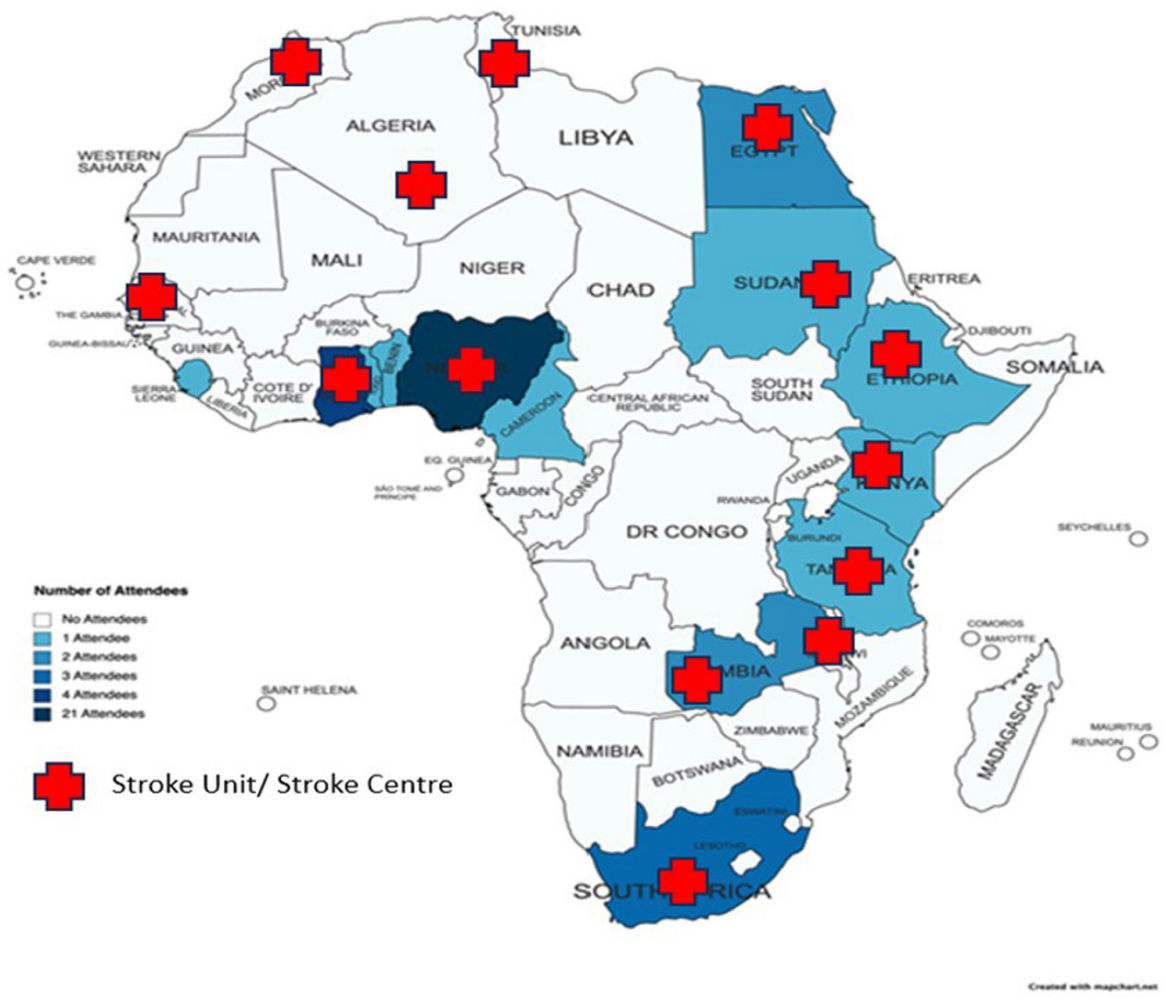
The participating African countries.

A total of 52 stroke experts participated in the first African Leaders' Stroke Summit. They represented 13 African countries (Figure 2), the UK, Sweden, and the USA. The professional background covered a wide spectrum of expertise including neurology, geriatrics, neurosurgery, stroke nursing, neurophysiology, physiotherapy, psychology, health education and care, radiology, neuropathology, stroke survivor, and a policymaker. The policy maker was a past minister of health of the Federal Republic of Nigeria.

### 2.3 Sessions

The ASLS consisted of six sessions: Day 1: an opening session on the general overview of the burden of stroke in Africa and the objectives of the AUKSP Project were reviewed with two plenary sessions on the activities and findings by the WGs. Day 2: The Summit broke out into WGs to generate theme-based strategic priorities. The Summit concluded with a final consensus-building session to identify the five topmost priorities for implementation and generate feedback from the WGs. The session also conducted a poll on the topmost priorities for action for each theme (Table 2).

**Table 2:** Priorities identified in the WGs.

**Working group**	**Top five strategies during the breakout session**
Stroke services	•Population-based stroke education including focus on primary stroke prevention, detection and treatment of risk factors
	•Promotion and development of acute stroke care through multidisciplinary teams and stroke units
	•Establishment of an African stroke care and quality commission within ASO
	•An integrated approach to improving access to stroke diagnostics
	•Integration of stroke care pathways, teams and referrals
Stroke advocacy	•Establishment of stroke advocacy priorities for research, services and capacity building
	•Increase attention to overall life after stroke for every stroke survivor
	•Involvement of people with lived experience of stroke and SSOs in setting research priorities
	•SSOs and patients groups should be recognized as equal partners across stroke care pathway in all African nations
	•African countries must have national stroke plans that address surveillance, prevention, treatment and rehabilitation informed by unique cultural, socioeconomic and geographic contexts
Stroke training	•Basic training to mitigate gross shortage of stroke experts including stroke neurologists, nurses and other professionals in Africa
	•Reperfusion therapy and multidisciplinary stroke care are most needed training programs
	•Cascade training e.g. to ensure nurse practitioners and staff have relevant knowledge and skills to perform their roles
	•Public awareness and education about stroke as a medical emergency
	•Stroke is better prevented, so primary prevention and secondary strategies should to be emphasized
Stroke research	•Research on hypertension control as a strategy for effective stroke control
	•Public awareness of stroke and stroke risk factors
	•Stroke services and implementation science research to inform practice and policy
	•Hospital- and community-based stroke surveillance
	•Basic discovery research to unravel novel therapeutics to prevent and treat stroke with potential benefit for the global community

A Google poll was designed and distributed electronically among the participants in the ASLS. The poll questionnaire consisted of 20 distinct items based on the priorities generated during the WG sessions (Table 2). Participants rated the priorities within each theme on a Likert scale of 1 to 5, where 1 represented the least and 5 signified the highest priority. The item from each domain with the highest total score was selected as the topmost priority for each of the themes.

## 3 Results

### 3.1 Implementation gaps highlighted in the situational analysis reports from the WGs

The implementation gaps identified in the situational analysis reports based on the four themes and undertaken by the four corresponding WGs in preparation for the ASLS are summarized as follows.

#### 3.1.1 Stroke services

##### 3.1.1.1 Population-based stroke services (surveillance and primary prevention)

There are varied implementation plans with uneven progress across Africa. Some countries have reported surveillance studies across multiple sites (Akinyemi et al., 2021a). Although some countries have nutritional guidelines for non-communicable disease (NCD) prevention and control, national plans for routine, regular, or periodic stroke surveillance are lacking. The availability of essential medications at primary healthcare centers (PHCs) is challenging in multiple countries. High level of out-of-pocket (OOP) expenditures are prevalent across the African countries surveyed coupled with the paucity of national health insurance programmes. This poses a huge financial barrier to accessing effective stroke care and adherence to preventive medications. By 2030, strategic initiatives to reduce the stroke burden should be encouraged within respective African countries and such initiatives should focus on key lifestyle areas such as alcohol use, tobacco smoking, food choices, blood pressure management, fruit and vegetable intake, childhood malnutrition, school feeding programmes, and diabetes management. Respective African countries should work toward developing a national stroke action plan, national policy on NCDs (including guidelines on their prevention and control), and stroke registries. National or regional pilot m-Health programs for stroke risk factor reduction and control should be rapidly developed.

##### 3.1.1.2 Pre-hospital/emergency stroke services

According to a 2017 study, 16 African countries had an Emergency Medical Services (EMS) system or an EMS 'hotline'. EMS training is well-established in Southern Africa (Mould-Millman et al., 2017). Hospital ambulance services were available in 56% of the African countries surveyed (Mould-Millman et al., 2017). The African Federation of Emergency Medicine (AFEM) has published operational recommendations for EMS services for sub-Saharan Africa (Calvello et al., 2016). National efforts should be made to promote the routine use of EMS performance measures for acute stroke care and increase awareness of the association between EMS and stroke outcomes in all African countries by 2030.

##### 3.1.1.3 Hospital-based acute stroke services

The acute stroke care landscape is heterogeneous, particularly across sub-Saharan Africa (SSA). Significant barriers limit access to neuroimaging (such as cost and geographic penetration within countries) even though Roushdy et al. (2022), had observed that CT scanning was available across all the 17 countries. There is a paucity of stroke units, and reperfusion therapies such as endovascular therapies are limited by high cost and manpower shortages. To further improve stroke care in Africa, coordinated multidisciplinary stroke care should be promoted, ideally in a dedicated stroke unit in all African countries. Cadilhac et al. (2011) suggest that facilities evaluating more than 100 stroke cases per year should prioritize having stroke units. The personnel needs to be qualified as regards the acuteness of stroke. However, feasibility, cost, and staffing implications in chronically under-staffed, and poor-resourced settings are realistic challenges. All stroke patients should, at the minimum, be seen by a coordinated multidisciplinary team even in situations where formal stroke units are not available.

##### 3.1.1.4 Stroke recovery and rehabilitation services

In-hospital stroke rehabilitation is commonly available in many African countries but there are significant barriers to outpatient and community-based rehabilitation (Urimubenshi et al., 2018). There is poor access to community rehabilitation in some countries (Baatiema et al., 2017b) compared to others (Wasserman et al., 2009; Scheffler and Mash, 2019). Ideally, all stroke patients should have access to early rehabilitation services, tailored to their needs, by expanding the delivery of rehabilitation services in the community using multiple approaches including m-health, tele-physiotherapy, and task shifting given that stroke is largely handled by a limited pool of neurologists in Africa.

#### 3.1.2 Stroke training/capacity building

The stroke care team comprises stroke specialists and non-specialist healthcare providers (Osuegbu et al., 2022). However, Bower and Zenebe (2005), reported a gross shortage of neurologists in Africa with no neurologists in some tertiary hospitals. Additionally, care within many settings is often poor and fragmented (Pandian et al., 2007; Baatiema et al., 2017a). When compared to high-income countries (HICs), there is a low uptake of thrombolytic therapy (Berkowitz et al., 2014). Better outcomes (lower death rates, reduced hospital stays) were recorded when a multi-disciplinary team managed patients in a stroke unit in South Africa by Villiers et al., and even in a non-stroke unit setting in Nigeria (Villiers et al., 2009; Adeniji et al., 2023). Also, in a study conducted in Morocco on thrombolytic therapy, almost 50% of the patients had a good outcome (Chtaou et al., 2016). A post-discharge functional independence of about 40% of patients after thrombolytic therapy was recorded in South Africa though complications occurred in about 12% (Von Klemperer et al., 2014; Bryer and Wasserman, 2013). Thrombolytic therapy is still not commonly available in many African countries (Baatiema et al., 2017b). PHCs/Community health facilities face huge staffing and funding challenges in Africa. Besides South Africa, there is unavailability of 24-h stroke services in many countries (Bryer et al., 2011). PHCs function as referral sources to tertiary hospitals but can potentially provide some care after discharge (Smythe et al., 2022). There are ongoing interventions targeted at training to improve nurse-led post-stroke care in Ghana and South Africa; otherwise, specialist nurse training for stroke is scarce across the continent. Instead, patients are typically nursed by generalist nurses on medical wards/units. However, evidence from the region suggests specialized stroke nurses could lead to demonstrable gains in patient outcomes. In a randomized controlled trial of a nurse-led m-health post-stroke intervention for blood pressure (BP) control in Ghana, there was a significant reduction in BP and high levels of medication adherence in the intervention arm (Sarfo F. et al., 2018). The identified roles of South African nurses in the rehabilitation of patients admitted for acute stroke include clinical care service provision, emotional support, and provision of rehabilitation services. This consists of patient mobilization, therapeutic positioning, exercises, and the teaching of basic self-care besides communication skills (Mhango, 2018). Early involvement of physiotherapists during the acute phase is not always feasible due to staffing shortages leading to poor outcomes in some parts of Africa compared to the rest of the world (Akinyemi et al., 2021a).

Therefore, personnel at all PHCs and private clinics should be trained with adequate resources to diagnose and triage patients with stroke. All PHCs must be strengthened for acute stroke care and post-stroke rehabilitation services across communities through access to care by a stroke specialist nurse, and rehabilitation by a physiotherapist.

#### 3.1.3 Stroke research

Output of stroke research in Africa has increased over the past four decades even though there is still room for improvement (Akinyemi et al., 2021a). Most of the publications were epidemiological studies either (cross-sectional, or case-control) while clinical trials across the stroke care continuum and implementation science research (adapting, testing, and scaling up proven interventions) are limited in scope (Owolabi et al., 2021). There is, thus, an urgent priority to move from a description of the burden of stroke in Africa to designing and testing interventions that will improve outcomes. Stroke services research including clinical audits and quality of care studies are urgently needed to inform policy and resource allocation. Regionalized platforms such as the African Stroke Organization Conference meet a critical need of providing a venue for contributions arising from scholars of African descent (Gebreyohanns et al., 2020, 2022). Basic research including genomics and multi-omics studies in Africa may unveil novel diagnostics and therapeutics for prevention, diagnosis, treatment, and prognosis with potential benefit for the global community (Akinyemi et al., 2015; Owolabi et al., 2017). Local, regional, and national stroke registries are lacking in many African countries while local projects on stroke epidemiology, acute care, secondary prevention, and rehabilitation are unevenly distributed. Stroke population science research, including epidemiological surveillance (incidence, prevalence, outcomes, risk factors), is available in some regions, but more is needed to inform strategic plans. Studies on life after stroke and stroke outcomes research are crucial. In order to achieve these by 2030, there should be firm national commitments to the establishment and maintenance of national and regional stroke registries. There has to be a translation of proven population- and individual-level stroke preventive strategies through implementation research that investigates the efficacy of incorporating technologies such as the use of mobile phones and telehealth into stroke prevention, acute care, and rehabilitation services is imperative. In addition, stroke genomics research must be upscaled to unveil and/or clarify novel genetic determinants of stroke risk, and stroke severity in Africans in order to identify the genetic, biochemical, imaging, and other biomarkers of increased stroke risk and determinants of stroke outcomes. Such discoveries will potentially preface the development of effective preventive and therapeutic strategies targeting such biomarkers and plausibly chart the course for personalized interventions. Identification of effective, culturally acceptable, economically feasible, contextually relevant evidence-based interventions for stroke is germane and of paramount importance in Africa.

#### 3.1.4 Stroke awareness and advocacy

African SSOs are heterogeneous in scale and scope, and the WSO global mapping of SSOs demonstrated that SSOs are raising awareness of stroke among the general population and supporting patients, family, and carers across the stroke care pathway, yet SSOs access low levels of government funding in sub-Saharan Africa (SSA) (Lindsay et al., 2019). One of the most important factors to determine health-seeking behavior is awareness and knowledge of the disease; as well as attitude toward stroke as a medical condition. Some African countries either have no word for stroke, or only recently adopted terminology specific to this pathology; there is low knowledge and awareness of cerebrovascular diseases, their risk factors, signs, and symptoms (Aseffa et al., 2019). Knowledge level varies by population type, place of residence, employment status, educational attainment, and religious and cultural belief (Boateng et al., 2017). Awareness of established stroke signs and symptoms ranged between 18% in a Uganda study and 66% in Nigeria while between 1% and 13% of survey participants identified seeking spiritual intervention as the first treatment choice (Urimubenshi et al., 2018). A lack of culturally compatible content may contribute to misunderstanding of people living with stroke resulting in delayed medical care, particularly among the young who believe that stroke is a disease of the elderly (Louw et al., 2020; Beauchamp et al., 2022; Zewdu et al., 2020). In the Northern African and Middle East region, strong leadership, improved government funding, enhanced stroke awareness, training, and partnerships have transformed acute stroke care (Akinyemi et al., 2021a; Mohammed et al., 2020). The INTERSTROKE Study documented a lack of knowledge that hypertension causes stroke such that there were associated lower rates of any lifetime blood pressure measurement and non-use of antihypertensive therapy in those with known hypertension and associated higher rates of intracerebral hemorrhage in low and middle-income countries (LMIC)/low-income countries (LICs) than HICs (O'Donnell et al., 2021). In the SIREN Study, the major theme across all groups regarding the cause of stroke at the individual level centered around “*stress”* (Jenkins et al., 2018). In a community study in Ghana, only 40% of 693 respondents correctly identified the brain as the organ affected by stroke, and less than half of respondents could recognize any established stroke risk factors or warning signs (Donkor et al., 2014). In Nigeria, adults recognized hypertension as the most common risk factor for stroke better than adolescents. However, adolescents recognized weakness as the most common sign of stroke better than adults did (Komolafe et al., 2015). By 2030, NCD national strategies should be developed to include: (1) Stroke prevention, treatment, and support, (2) Interventions to increase awareness, screening, and control of the leading risk factors for NCDs, including, hypertension, dyslipidaemia, diabetes mellitus; and (3) Patient and community organizations as key stakeholders and strengthening the capacity of SSOs. Contextually appropriate national campaigns should be funded and implemented to promote understanding of stroke, support healthy lifestyles, and risk factor management. Moreover, there should also be a focus on post-stroke rehabilitation and life after-stroke issues including return to work/occupation, housing, and palliative care.

### 3.2 Priorities identified by the working group breakout sessions

Table 2 presents the outcomes of the WG breakout sessions. Each WG generated a list of five priorities considered necessary to advance the science and care of stroke in Africa.

### 3.3 Consensus building session: results of polling exercise

**Figure 3:**
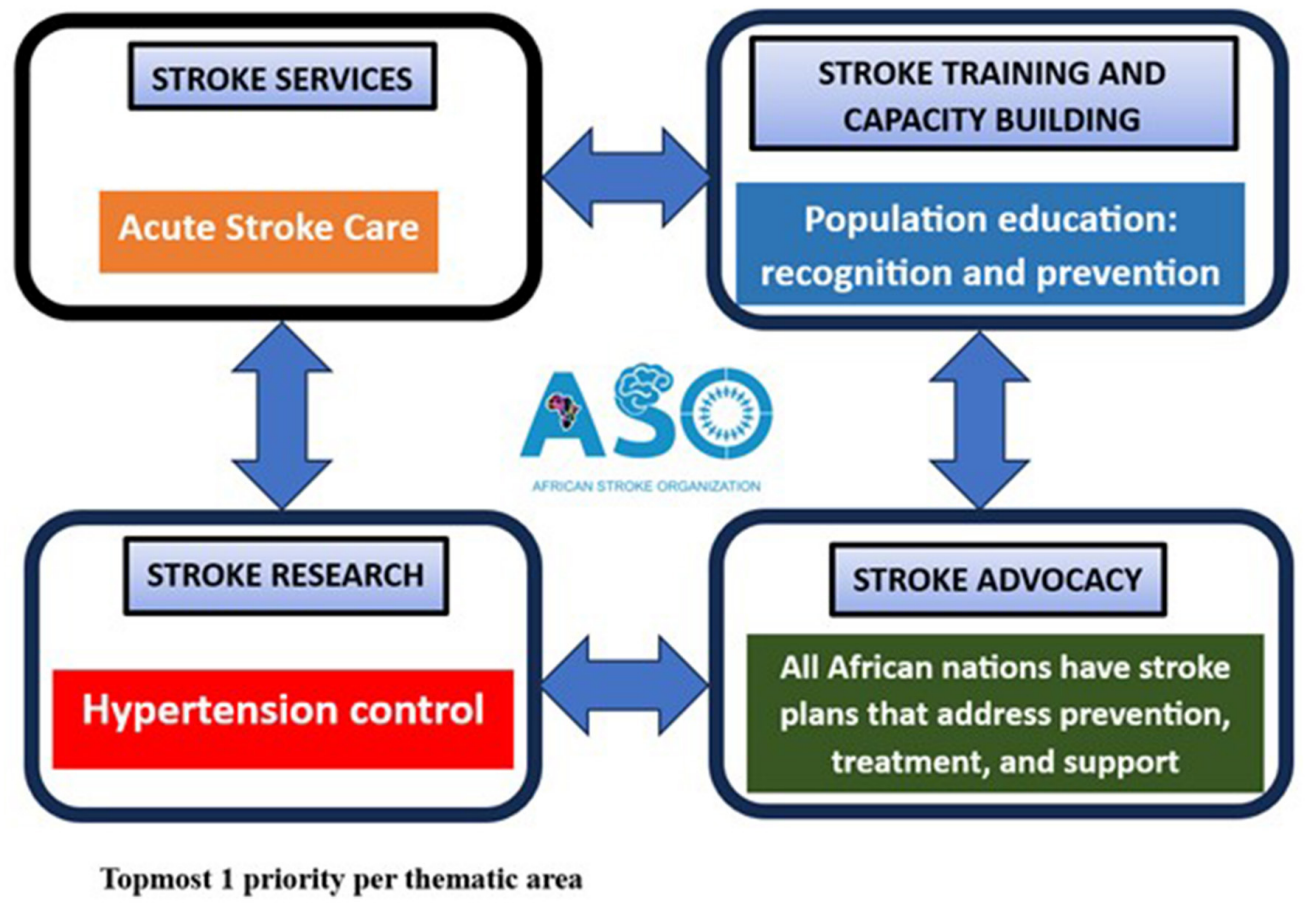
Topmost 1 priority per thematic area.

Figure 3 represents the outcomes of the polling activity in each thematic area. The topmost 1 priority action steps generated by the WGs (Table 2) following the analysis of the results of the poll are displayed.

## 4 Discussion

The purpose of AUKSLS was to identify gaps and priorities for improving stroke services, advocacy, training, and research in Africa. Theme–based WGs conducted a situational analysis of the African stroke landscape to generate 20 priorities (five per theme) during the WG sessions. These were refined to four topmost priorities following a consensus poll undertaken by the participants. The topmost priorities included promoting the development of acute stroke services, developing national stroke action plans, strengthening population-based stroke education focusing on prevention and symptom recognition, and research on hypertension control to reduce stroke risk.

As reported previously, the acute stroke services are uneven with few acute stroke units in many SSA nations. Reperfusion therapies such as mechanical thrombectomy and thrombolysis are even less available and affordable. Strategies to mitigate the current challenges require multipronged approaches that include willingness and capacity to learn from one another in Africa. Notable initiatives to improve acute stroke care services include the Angels Initiative (Caso et al., 2018), and the Wessex-Ghana Stroke Partnership (Johnson et al., 2017) both of which have demonstrated promising success. A University hospital-led initiative in Egypt to improve adoption of thrombolysis for acute stroke spurred strong government support to provide reimbursement for IV thrombolysis usage (Zakaria et al., 2018). This initiative was a strong incentive for adoption of stroke units and thrombolysis across Egypt with an estimated 95 stroke units established by 2020 (Aref et al., 2021). African governments need to provide support at national and regional levels to develop stroke services offering essential and advanced stroke services in line with global best practices and when it is not feasible to have a dedicated facility for an acute stroke unit, minimal stroke units (Lindsay et al., 2014) or multidisciplinary stroke teams (Adeniji et al., 2023). The development of pre-hospital stroke services, including emergency ambulance services are also necessary for growing acute stroke services.

The topmost priority identified for the stroke advocacy theme was the development of national stroke action plans covering every aspect of stroke care continuum, including prevention, surveillance, acute care, rehabilitation, and life after stroke. Although seven African countries have a national stroke action plan, they differ in quality and content. A national stroke action plan should be a policy document that describes the strategic and contextual framework of the country's action roadmap toward a stroke–free society. It is a policy blueprint that should describe access to human resources and infrastructure as well as a plan of action for future development for stroke care, research, and capacity- building. The ASO has every intent to work with the Lancet WSO Stroke Commission Stroke to stimulate relevant development of national stroke action plans across African nations.

For the stroke training/capacity building theme, population–based stroke education with emphasis on prevention and symptom recognition was highlighted as priority. Previous studies have explored knowledge and perception of stroke in Africa but found largely suboptimal awareness and perceptions influenced by religious and cultural beliefs (Akinyemi et al., 2021a). A systematic review identified low levels of knowledge and awareness of cardiovascular diseases (CVDs), risk factors, and signs or symptoms in SSA. There was also a low perception of the risk of developing and dying from CVDs (Boateng et al., 2017). It is imperative to develop and scale up educational stroke–specific programmes using contextually appropriate strategies and approaches.

Research on hypertension awareness, treatment, and control as a strategy for stroke risk reduction is rated topmost in Africa. Although, hypertension remains the major modifiable risk factor for strokes in Africa, and has a prevalence of nearly 50% with 93% of cases uncontrolled, very little progress has been made in detecting, treating, and controlling hypertension (Owolabi et al., 2023). A recent cohort study of over 3,000 outpatients with hypertension at five public hospitals in Ghana (Sarfo F. S. et al., 2018) found that male sex, low levels of education, non-adherence to antihypertensive medications, low fruit intake, comorbid diabetes, long duration of hypertension diagnosis, number of hypertensive medications and kidney disease were predictors of poor BP control over the study period. These findings highlight high-risk groups for poor hypertension control. Other factors hindering hypertension control from studies include negative primary care experience, poor social support, lack of awareness, and high financial barriers to accessing drugs (Sarfo F. S. et al., 2018). The African Control of Hypertension through Innovative Epidemiology and a Vibrant Ecosystem (ACHIEVE) initiative was commissioned as a special African initiative to promote the HEARTS package for improved surveillance, prevention, treatment/acute care, and rehabilitation of those with hypertension complications across the life course (Owolabi et al., 2023). Effective control of hypertension and other risk factors will drastically reduce the burden of stroke in Africa.

## 5 Limitations

Only a fraction of the 54 African countries were represented, and this may reflect the dearth of stroke care capacity in many African countries.

## 6 Conclusions and future directions

Translating research evidence into practice and policy is an enormous challenge in many stroke care systems around the world and in Africa. However, substantial gaps remain in the full understanding of stroke and in improving stroke care, practice, and policy. Building a critical mass for change and sustaining improvements is urgent. It is necessary to forge local and international partnerships, adapt best practices to suit local environment, and build capacity among African stroke care professionals to champion the change in stroke care and, use available resources. Political will, managerial buy-in, and clinical leadership at the macro-, meso- and micro-levels are essential to creating, encouraging, and sustaining a climate where these changes become possible. Further, improving on information on stroke services and communicating needs of African communities and countries would be a complementary strategy to reinforce healthy lifestyle choices, improve screening and detection of stroke risk factors. The time to take action is now. This report is a landmark in the process of developing a strategic African Stroke Action Plan (ASAP), the blueprint for the control of stroke in Africa.

## Data Availability

The datasets presented in this article are not readily available. Requests to access the datasets should be directed to Rufus Akinyemi, rufusakinyemi@yahoo.com.
